# Synergy between inhibitors of androgen receptor and MEK has therapeutic implications in estrogen receptor-negative breast cancer

**DOI:** 10.1186/bcr2858

**Published:** 2011-04-01

**Authors:** Ali Naderi, Kee Ming Chia, Ji Liu

**Affiliations:** 1 The University of Queensland Diamantina Institute, Princess Alexandra Hospital, Ipswich Road, Brisbane, Queensland 4102, Australia

## Abstract

**Introduction:**

Estrogen receptor-negative (ER-) breast cancer is a heterogeneous disease with limited therapeutic options. The molecular apocrine subtype constitutes 50% of ER-tumors and is characterized by overexpression of steroid response genes including androgen receptor (AR). We have recently identified a positive feedback loop between the AR and extracellular signal-regulated kinase (ERK) signaling pathways in the molecular apocrine subtype. In this feedback loop, AR regulates ERK phosphorylation through the mediation of ErbB2 and, in turn, ERK-CREB1 signaling regulates the transcription of AR in molecular apocrine cells. In this study, we investigated the therapeutic implications of the AR-ERK feedback loop in molecular apocrine breast cancer.

**Methods:**

We examined a synergy between the AR inhibitor flutamide and the MEK inhibitor CI-1040 in the molecular apocrine cell lines MDA-MB-453, HCC-1954 and HCC-202 using MTT cell viability and annexin V apoptosis assays. Synergy was measured using the combination index (CI) method. Furthermore, we examined *in vivo *synergy between flutamide and the MEK inhibitor PD0325901 in a xenograft model of the molecular apocrine subtype. The effects of *in vivo *therapies on tumor growth, cell proliferation and angiogenesis were assessed.

**Results:**

We demonstrate synergistic CI values for combination therapy with flutamide and CI-1040 across three molecular apocrine cell lines at four dose combinations using both cell viability and apoptosis assays. Furthermore, we show *in vivo *that combination therapy with flutamide and MEK inhibitor PD0325901 has a significantly higher therapeutic efficacy in reducing tumor growth, cellular proliferation and angiogenesis than monotherapy with these agents. Moreover, our data suggested that flutamide and CI-1040 have synergy in trastuzumab resistance models of the molecular apocrine subtype. Notably, the therapeutic effect of combination therapy in trastuzumab-resistant cells was associated with the abrogation of an increased level of ERK phosphorylation that was developed in the process of trastuzumab resistance.

**Conclusions:**

In this study, we demonstrate *in vitro *and *in vivo *synergies between AR and MEK inhibitors in molecular apocrine breast cancer. Furthermore, we show that combination therapy with these inhibitors can overcome trastuzumab resistance in molecular apocrine cells. Therefore, a combination therapy strategy with AR and MEK inhibitors may provide an attractive therapeutic option for the ER-/AR+ subtype of breast cancer.

## Introduction

Estrogen receptor-negative (ER-) breast cancer constitutes around 30% of all cases with limited therapeutic targets available for this heterogeneous disease [1]. In contrast to ER+ breast cancer, in which anti-estrogen therapy is an effective treatment strategy, current therapeutic options for advanced ER-breast cancer mostly rely on chemotherapeutic agents.

Molecular profiling of ER-breast cancer broadly classifies this disease into basal and molecular apocrine subtypes [2]. Molecular apocrine breast cancer constitutes approximately 50% of ER-tumors and is characterized by a steroid response gene signature that includes androgen receptor (AR) and a high frequency of ErbB2 overexpression [2-8]. For pathological classification, this subtype can easily be characterized as ER-/AR+ breast cancer [6-8]. In a recent study by Park *et al*. [7], AR expression was observed in 50% of ER-breast tumors and in 35% of triple-negative cancers. In addition, ErbB2 overexpression was present in 54% of ER-/AR+ tumors compared to 18% of the ER-/AR-group, which suggests a significant correlation between AR expression and ErbB2 overexpression in ER-tumors [7]. Importantly, a growing body of evidence suggests that AR is a therapeutic target in molecular apocrine breast cancer [4,5,9]. In this regard, AR inhibition reduces cell viability and proliferation in molecular apocrine models [4,5,9]. In addition, an ongoing clinical trial has demonstrated that AR inhibition can stabilize disease progression in metastatic ER-/AR+ breast cancer [10].

AR signaling has a significant role in the biology of molecular apocrine tumors. Notably, we have identified a functional cross-talk between the AR and ErbB2 signaling pathways in molecular apocrine cells that modulates cell proliferation and expression of steroid response genes [5]. In addition, this cross-talk has been confirmed by a genome-wide meta-analysis study [11]. Moreover, we have recently discovered a positive feedback loop between the AR and extracellular signal-regulated kinase (ERK) signaling pathways in molecular apocrine breast cancer [12]. In this feedback loop, AR regulates ERK phosphorylation through the mediation of ErbB2, and, in turn, ERK-CREB1 signaling regulates the transcription of AR in molecular apocrine cells [12].

The AR-ERK feedback loop has potential therapeutic implications in molecular apocrine breast cancer. In particular, due to the availability of effective AR and mitogen-activated protein kinase kinase (MEK) inhibitors, exploiting this feedback loop would provide a practical therapeutic approach. A number of AR inhibitors are currently used for prostate cancer, and their safety in a female patient population has been demonstrated in studies of breast and ovarian cancers [10,13,14]. Furthermore, several classes of MEK inhibitors have been developed and are now being examined in various clinical trials [15,16]. Therefore, a potential positive outcome for the preclinical studies can readily be tested in future clinical trials.

Here we carried out a preclinical study of combination therapy with AR and MEK inhibitors using *in vitro *and *in vivo *molecular apocrine models. Our results suggest that this combination therapy provides a promising therapeutic strategy in ER-/AR+ breast cancer.

## Materials and methods

### Cell culture and treatments

Breast cancer cell lines MDA-MB-453, HCC-202, and HCC-1954 were obtained from the American Type Culture Collection (Manassas, VA, USA). All the culture media were obtained from Invitrogen (Melbourne, VIC, Australia). MDA-MB-453 cell line was cultured in L15 media/10% fetal bovine serum (FBS). HCC-202 and HCC-1954 cells were cultured in RPMI 1640 media with 10% FBS. Cell cultures were carried out in a humidified 37°C incubator supplied with 5% CO_2_. The following treatments were applied for the cell culture experiments: (1) AR inhibitor flutamide (Sigma-Aldrich, Sydney, NSW, Australia) at 5 to 200 μM concentrations; (2) MEK inhibitor CI-1040 (PD184352) (Selleck Chemicals, Houston, TX, USA) at 2 to 30 μM concentrations; and (3) ErbB2 inhibitor trastuzumab (Roche, Sydney, NSW, Australia) at 10 to 80 μg/ml concentrations. Treatments with the inhibitors were performed in media containing FBS.

### Cell viability assay

MDA-MB-453, HCC-202 and HCC-1954 cells were grown in 96-well plates to 50% confluence followed by inhibitor treatments for 48 hours in full media. A solvent-only-treated group was used as a control. Cell viability was assessed using the Vybrant MTT Proliferation Assay Kit (Invitrogen) as previously described [5,17]. Absorbance at 570 nm was measured for the experimental groups using a plate reader. MTT experiments were performed in eight biological replicates.

### Apoptosis assay

Apoptosis measurement with flow cytometry was carried out using Annexin V-FITC Apoptosis Detection Kit I (BD Biosciences, Sydney, NSW, Australia). All experiments were performed in four biological replicates.

### Combination indices

Drug synergy was assessed using a combination index (CI) method as described before [9,18]. We first measured cell viability and apoptosis for the combination therapies with flutamide and CI-1040 using MTT and annexin V assays, respectively. We next identified the concentrations of flutamide and CI-1040 monotherapies, which resulted in a level of reduction in cell viability and apoptosis similar to that observed with each of the combination therapy conditions. Subsequently, CI for the combined treatments were calculated as follows: CI = [Ca,*x*/IC*x*,a] + [Cb,*x*/ICx,b], Ca,*x *and Cb,*x *are the concentrations of drug A and drug B used in combination to achieve *x*% drug effect [18]. IC*x*,a and IC*x*,b are the concentrations for single agents to achieve the same effect. A CI less than 1 indicates synergy with the combination therapy.

### Tumor xenograft studies

Animal ethics approval was obtained for the project, and mice were maintained in accordance with the Institutional Animal Care guidelines. Six-week-old female nonobese diabetic/severe combined immunodeficient mice were purchased from Animal Resource Center (Perth, WA, Australia). The methodology for generating the tumors in mice was performed as previously described [9,12]. A total of 5 × 10^6 ^MDA-MB-453 cells were injected into the flank of each mouse to generate the xenograft tumors [9]. Drug treatments were initiated 7 days after the cell injections.

Flutamide treatment was carried out with 25 mg/60-day slow-release flutamide pellets (Innovative Research of America, Sarasota, FL, USA), and the control group received placebo pellets (Innovative Research of America). MEK inhibitor treatment was carried out with daily oral gavage of PD0325901 (Selleck Chemicals) at 5 to 20 mg/kg/day as described before [19]. PD0325901 was prepared at a stock concentration of 50 mg/ml in dimethyl sulfoxide (DMSO) (Sigma-Aldrich) and made up to the daily working concentration in 0.05% methylcellulose/0.02% Tween 80 (Sigma-Aldrich). The control group received daily gavage of a volume of DMSO equal to that of the treatment group in the same carrier solution.

The tumor volumes were assessed every 3 days by measuring the length (*l*) and width (*w*) and then calculating the volume as π/6 × *l *× *w *× (*l *+ *w*)/2 as described before [20]. Xenograft tumors were harvested 30 days following the start of treatments. Fold change in tumor volume was calculated as [volume on treatment day 30/volume on treatment day 1]. Harvested tumors were fixed in formalin and embedded in paraffin for immunohistochemistry (IHC) staining.

### Toxicity studies in mice

We assessed toxicity to MEK inhibitor in mouse xenograft model by measuring body weight change during 30 days of treatment with PD0325901 at 5 to 20 mg/kg/day. The control group received daily gavage of carrier solution. Xenograft experiments were carried out as explained before, and two mice were treated per each treatment group. Mice were weighed daily during the course of treatment. In the event of weight reduction for two consecutive days, drug was withheld until weight stabilized before therapy reinitiation. Toxicity was evaluated by the measurement of (1) weight change pre- and post-treatment in each group and (2) number of treatment days lost due to weight reduction or mortality.

### Immunohistochemistry

IHC staining was performed using EnVision+ System-HRP (AEC, DakoCytomation, Melbourne, VIC, Australia) following the manufacturers' instruction. Antigen retrieval was carried out using Target Retrieval Solution (DakoCytomation). Rabbit polyclonal Ki-67 and rabbit polyclonal CD31 antibodies were obtained from Abcam (Cambridge, UK). Primary antibody incubation was carried out at 1:50 dilution for each antibody. Slides were counterstained with hematoxylin (Sigma-Aldrich) and mounted using Glycergel Mounting Medium (DakoCytomation). For IHC scoring, slides were examined using a light microscope at ×60 magnification (Nikon Instruments Inc., Tokyo, Japan).

The percentage of cells showing Ki-67 nuclear staining in a total of 600 cells was calculated as the proliferation index for each tumor. The total number of CD31-positive blood vessels in a tumor cross-section was counted to measure angiogenesis in each sample. Scoring was carried out separately by two investigators, and the average scores were used for the final analysis.

### Generation of trastuzumab-resistant line

To generate a trastuzumab-resistant line, MDA-MB-453 cells were continuously cultured with increasing doses of trastuzumab at 10 to 20 μg/ml concentrations for 90 days. The MDA-MB-453 control line was treated with solvent only and grown for the same duration. Cell viability of resistant and control lines were assessed using MTT assay.

### Western blot analysis

Rabbit monoclonal ERK1/2 and phospho-ERK1/2 (Thr202/Tyr204) antibodies were obtained from Cell Signaling Technology (Danvers, MA, USA). Western blot analysis was carried out at 1:1,000 dilution of each primary antibody using 10 μg and 20 μg of cell lysates for total and phospho-ERK1/2, respectively. Protein concentrations from the cell isolates were measured using BCA Protein Assay Kit (Thermo Scientific, Melbourne, VIC, Australia). Rabbit polyclonal α-tubulin antibody (Abcam) was used as loading control. Analysis of band densities was performed using Bio-Profil Densitometer Software (Vilber Lourmat, Eberhardzell, Germany). Fold changes in band densities were measured relative to the control groups. Western blot analysis was done in two biological replicates, and the average fold change was shown for each set of experiments.

### Statistical analysis

Biostatistical analysis was done using the SPSS version 17.0 statistical software package (SPSS, Inc., Chicago, IL, USA). The Mann-Whitney *U *test was applied for the comparison of nonparametric data.

## Results

### Synergy between AR and MEK inhibitors in reducing cell viability

**Figure 1:**
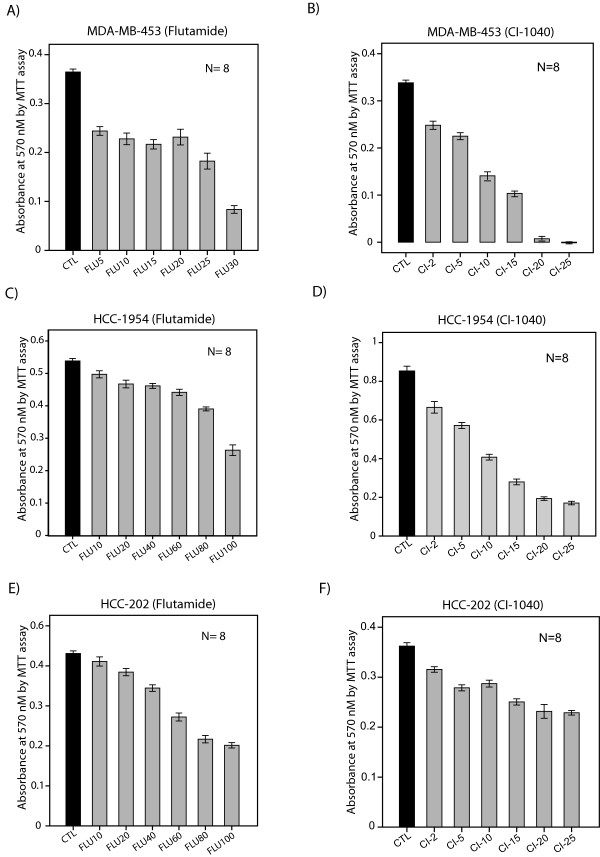
**The effect of flutamide and CI-1040 on cell viability of molecular apocrine lines**. **(A) **MTT assay to measure cell viability in MDA-MB-453 cell line after treatment with flutamide (FLU) at 5 to 30 μM concentrations. CTL: control. **(B) **MTT assay to measure cell viability in MDA-MB-453 cell line after treatment with CI-1040 (CI) at 2 to 25 μM concentrations. **(C) **MTT assay to measure cell viability in HCC-1954 cell line after treatment with flutamide at 10 to 100 μM concentrations. **(D) **MTT assay to measure cell viability in HCC-1954 cell line after treatment with CI-1040 at 2 to 25 μM concentrations. **(E) **MTT assay to measure cell viability in HCC-202 cell line after treatment with flutamide at 10 to 100 μM concentrations. **(F) **MTT assay to measure cell viability in HCC-202 cell line after treatment with CI-1040 at 2 to 25 μM concentrations. All error bars: ± 2 SEM.

**Figure 2:**
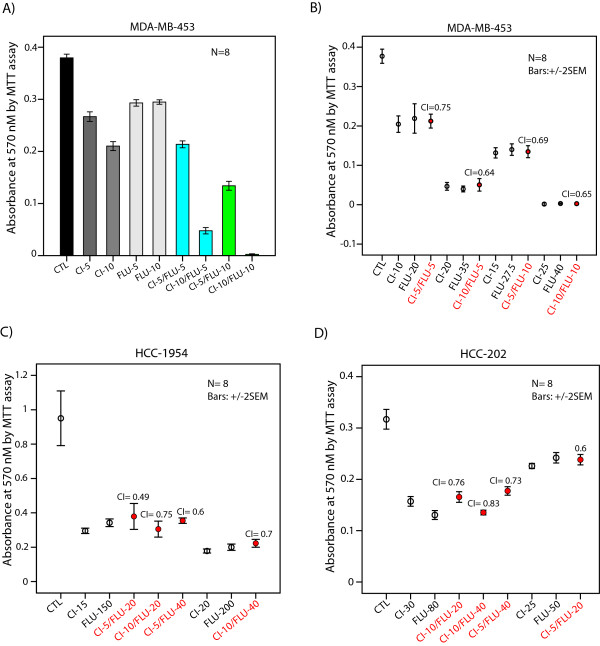
**Synergistic effect of AR and mitogen-activated protein kinase kinase inhibitors on cell viability**. **(A) **MTT assay to measure cell viability in MDA-MB-453 cell line after monotherapies and combination treatments with flutamide (FLU) and CI-1040 (CI) at 5 and 10 μM concentrations. CTL: control. Error bars: ± 2 SEM. **(B) **Combination indices (CI) for flutamide and CI-1040 combination therapy in MDA-MB-453 cell line using MTT assay. Cell viability was measured after combination therapies with flutamide at 5 and 10 μM with each concentration of CI-1040 at 5 and 10 μM. The concentrations of FLU and CI-1040 monotherapies with an effect similar to that of each combination therapy are depicted. Error bars: ± 2 SEM. **(C) **Combination indices for flutamide and CI-1040 combination therapy in HCC-1954 cell line using MTT assay. Cell viability was measured after combination therapies with flutamide at 20 and 40 μM with each concentration of CI-1040 at 5 and 10 μM. **(D) **Combination indices for flutamide and CI-1040 combination therapy in HCC-202 cell line using MTT assay at concentrations described in Figure 2C.

To assess a potential synergy between the AR inhibitor flutamide and the MEK inhibitor CI-1040, we used previously characterized molecular apocrine cell lines MDA-MB-453, HCC-1954 and HCC-202 [5,9]. CI-1040 has been commonly used to examine the effects of MEK inhibition on cell lines, and therefore it was chosen for *in vitro *experiments in this study [21-23]. The effect of monotherapies with flutamide at 5 to 200 μM and CI-1040 at 2 to25 μM concentrations on cell viability of molecular apocrine lines was assessed by MTT assay. We observed that monotherapies with these inhibitors reduced cell viability in a dose-dependent manner across three cell lines (Figures 1A to 1F and 2A to 2D).

It is notable that MDA-MB-453 cells were relatively more sensitive to flutamide treatment compared to the HCC-1954 and HCC-202 lines. In MDA-MB-453 cells, flutamide at 30 μM concentration reduced cell viability by approximately 75% compared to control (Figure 1A). However, in HCC-1954 and HCC-202 cell lines, there was a 50% reduction in cell viability with flutamide at 100 μM concentration (Figure 1C and 1E). Furthermore, HCC-202 cells were relatively less sensitive to CI-1040 treatment compared to the other two cell lines. In this respect, CI-1040 at 25 μM concentration reduced cell viability by over 75% in MDA-MB-453 and HCC-1954 cells compared to an approximately 30% reduction in the HCC-202 line (Figure 1B, D and 1F).

Next, we calculated CI values for the combined therapy with flutamide and CI-1040 at four dose combinations in each cell line (Figure 2). In MDA-MB-453 cell line, which had a high level of sensitivity to flutamide, this drug was applied at 5 and 10 μM in combination with CI-1040 at 5 and 10 μM concentrations (CI-1040 (5 μM)/flutamide (5 μM), CI-1040 (10 μM)/flutamide (5 μM), CI-1040 (5 μM)/flutamide (10 μM), and CI-1040 (10 μM)/flutamide (10 μM)). In HCC-1954 and HCC-202 cell lines, flutamide at 20 and 40 μM concentrations was assessed for synergy in combination with CI-1040 at 5 and 10 μM concentrations (CI-1040 (5 μM)/flutamide (20 μM), CI-1040 (10 μM)/flutamide (20 μM), CI-1040 (5 μM)/flutamide (40 μM), and CI-1040 (10 μM)/flutamide (40 μM)). Importantly, we observed a synergy at all four dose combinations across three cell lines. In MDA-MB-453 cell line, CI values for the combination therapy with flutamide and CI-1040 were 0.64 to 0.75 (Figure 2B). Furthermore, in HCC-1954 and HCC-202 lines, CI values for the combination therapy were 0.49 to 0.75 and 0.6 to 0.83, respectively (Figure 2C and 2D). These data suggest that AR inhibitor flutamide and MEK inhibitor CI-1040 have synergy in reducing cell viability of molecular apocrine cell lines.

### Synergy between AR and MEK inhibitors in inducing apoptosis

To further investigate the synergy between flutamide and CI-1040, we assessed the effect of this combination therapy on apoptosis in molecular apocrine cell lines. Apoptosis was detected using annexin V assay and analyzed by flow cytometry. Using this approach, we calculated CI values for the combination therapy with flutamide and CI-1040 at four dose combinations in each cell line. CI-1040 was applied at 5 and 10 μM in combination with flutamide at 20 and 30 μM concentrations (CI-1040 (5 μM)/flutamide (20 μM), CI-1040 (10 μM)/flutamide (20 μM), CI-1040 (5 μM)/fluatmide (30 μM), and CI-1040 (10 μM)/flutamide (30 μM)).

**Figure 3:**
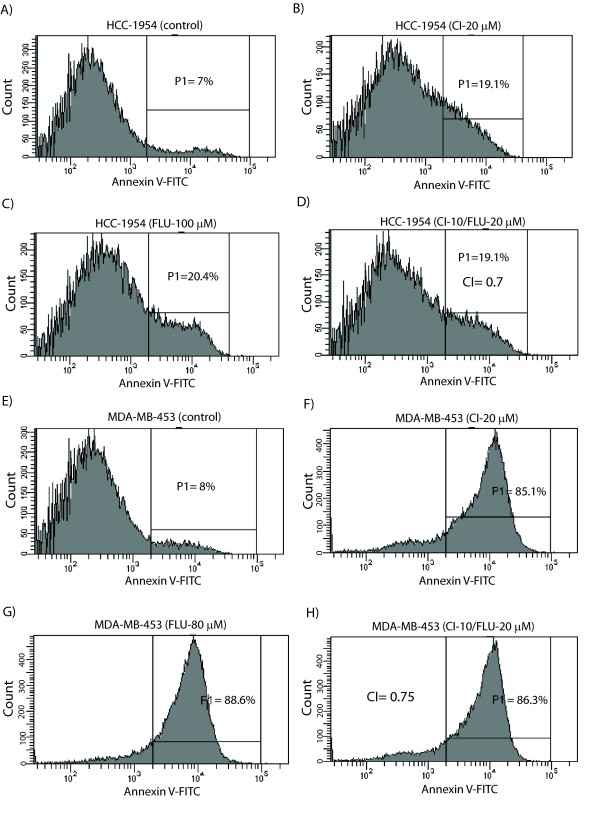
**Synergistic induction of apoptosis by AR and mitogen-activated protein kinase kinase inhibitors in HCC-1954 and MDA-MB-453 cell lines**. **(A) **Histogram showing the percentage of apoptosis (P1) in control (solvent-only treated) HCC-1954 cell line using annexin V-FITC flow cytometry. **(B) **Histogram showing the percentage of apoptosis following CI-1040 treatment at 20 μM (CI 20 μM) in HCC-1954 cell line. **(C) **Histogram showing the percentage of apoptosis following flutamide (FLU) treatment at 100 μM in HCC-1954 cell line. **(D) **Histogram showing the percentage of apoptosis following combination therapy with CI-1040 at 10 μM and flutamide at 20 μM in HCC-1954 cell line. Combination index (CI) is calculated using the concentrations of monotherapies with these agents as shown in Figures 3B and 3C that induced a level of apoptosis similar to that of combination therapy. **(E) **Histogram showing the percentage of apoptosis in control MDA-MB-453 cell line. **(F) **Histogram showing the percentage of apoptosis following CI-1040 treatment at 20 μM in MDA-MB-453 cell line. **(G) **Histogram showing the percentage of apoptosis following flutamide treatment at 80 μM in MDA-MB-453 cell line. **(H) **Histogram showing the percentage of apoptosis and CI following combination therapy with CI-1040 at 10 μM and flutamide at 20 μM in MDA-MB-453 cell line.

**Figure 4:**
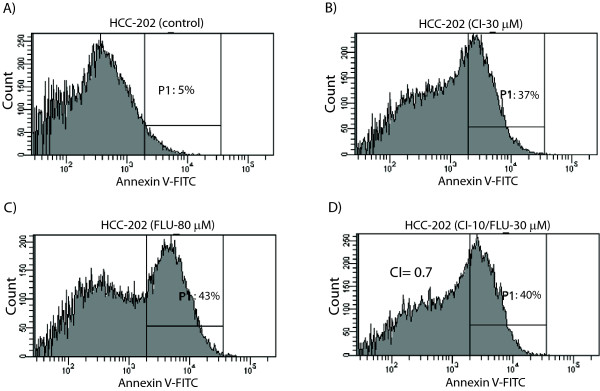
**Synergistic induction of apoptosis by AR and MEK inhibitors in HCC-202 cell line**. **(A) **Histogram showing the percentage of apoptosis (P1) in control (solvent-only treated) HCC-202 cell line using annexin V-FITC flow cytometry. **(B) **Histogram showing the percentage of apoptosis following CI-1040 treatment at 30 μM (CI-30 μM) in HCC-202 cell line. **(C) **Histogram showing the percentage of apoptosis following flutamide (FLU) treatment at 80 μM in HCC-202 cell line. **(D) **Histogram showing the percentage of apoptosis following combination therapy with CI-1040 at 10 μM and flutamide at 30 μM in HCC-202 cell line. CI is calculated using the concentrations of monotherapies with these agents as shown in Figures 4B and 4C that induced a level of apoptosis similar to that of combination therapy.

Notably, we observed synergy at all four dose combinations in molecular apocrine cell lines. In HCC-1954 and MDA-MB-453 cell lines, CI values for the combination therapy were 0.7 to 0.8 and 0.65 to 0.75, respectively (Figure 3A to 3H and Table 1). Furthermore, in the HCC-202 cell line, CI values for the combination therapy were 0.6 to 0.75 (Figure 4A to 4D and Table 1). Therefore, we can conclude that AR inhibitor flutamide and MEK inhibitor CI-1040 have synergy in the induction of apoptosis in molecular apocrine cell lines.

**Table 1:** Combination indices for apoptosis induced by flutamide and CI-1040 treatments

	Cell line					
	
Treatment	HCC-1954		MDA-MB-453		HCC-202	
CI-1040, μM	5	10	5	10	5	10
CI values FLU 20, μM	0.7	0.7	0.7	0.75	0.6	0.7
CI values FLU 30, μM	0.8	0.75	0.65	0.7	0.75	0.7

CI: combination index, FLU: flutamide. CI-1040 and flutamide concentrations are shown in μM.

### Assessment of MEK inhibitor toxicity in mice

We investigated the *in vivo *toxicity of PD0325901 to identify a tolerable dose of this MEK inhibitor for xeonograft studies. PD0325901 is a potent MEK inhibitor with chemical characteristics similar to that of CI-1040; however, a better oral bioavailability makes this agent more suitable for *in vivo *studies [19,24]. Following xenografts with MDA-MB-453 cells, mice were treated with daily oral gavage of PD0325901 at 5, 10, 15 and 20 mg/kg/day for 30 days. Daily gavage of carrier solution was used as control. Toxicity was evaluated by the measurement of weight change during treatment and number of treatment days lost due to weight reduction or mortality as described in Materials and methods.

**Figure 5:**
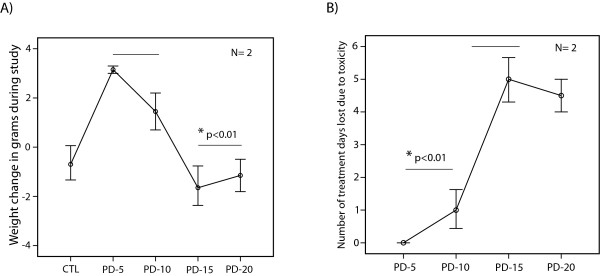
**Assessment of *in vivo *toxicity to MEK inhibitor PD0325901**. **(A) **Weight change in grams is shown for each PD0325901 (PD) treatment group in the MDA-MB-453 xenograft model. Weight change is the difference between pre- and post-treatment weight in each group. PD0325901 treatments were carried out at 5, 10, 15 and 20 mg/kg/day for 30 days, and daily gavage of carrier solution was used as control. *P < 0.01 for PD-5/PD-10 vs. control groups and PD-5/PD-10 vs. PD-15/PD-20 groups using Mann-Whitney *U *test. Error bars: ± 2 SEM. **(B) **Number of days lost due to toxicity is shown for each PD0325901 treatment group in mouse xenograft model explained in Figure 5A. *P < 0.01 for PD-5/PD-10 vs. PD-15/PD-20 groups.

We observed a significantly higher weight gain in mice treated with PD0325901 at 5 and 10 mg/kg/day doses compared to the control group (*P *< 0.01, Figure 5A). Importantly, treatments with higher doses of PD0325901 at 15 and 20 mg/kg/day resulted in a significant weight reduction compared to the lower doses of this agent (*P *< 0.01, Figure 5A). Furthermore, the number of treatment days lost due to toxicity was significantly lower with PD0325901 doses of 5 and 10 mg/kg/day compared to that of 15 and 20 mg/kg/day (*P *< 0.01, Figure 5B). Notably, PD0325901 treatment at 5 mg/kg/day did not result in any measurable toxicity using this approach (Figure 5A and 5B). These findings indicate that PD0325901 treatment at lower doses is significantly less toxic than higher doses of this agent in a xenograft mouse model.

### *In vivo *therapeutic efficacy of combination therapy with AR and MEK inhibitors

**Figure 6:**
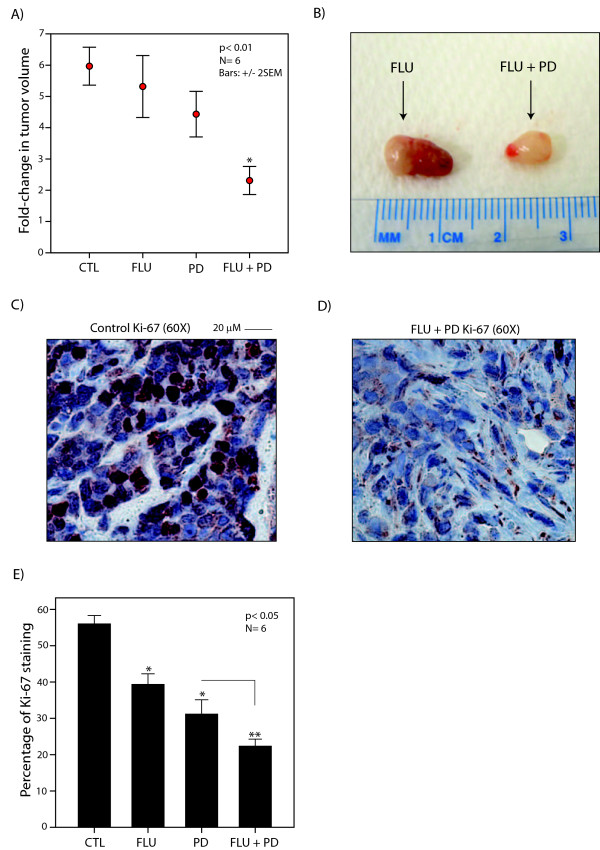
**The therapeutic effects of AR and MEK inhibitors on *in vivo *tumor growth and cellular proliferation**. **(A) **Fold change in tumor volume is shown for each *in vivo *treatment group using MDA-MB-453 xenograft model. CTL: control group; FLU: flutamide; PD: PD0325901. *P < 0.01 for the combination therapy group vs. control or monotherapy groups using Mann-Whitney *U *test. Error bars: ± 2 SEM. **(B) **Representative image of xenograft tumors in flutamide monotherapy and combination therapy groups. **(C) **Immunohistochemistry (IHC) was used to measure the proliferation index in a control xenograft tumor. Staining was carried out using a Ki-67 rabbit polyclonal antibody. Original magnification, ×60. **(D) **IHC was used to measure Ki-67 proliferation index in a xenograft tumor treated with the combination therapy. Original magnification, ×60. **(E) **Tumor proliferation indices using Ki-67 nuclear staining for the xenograft experiments. *P < 0.05 for monotherapy groups vs. control and **P < 0.05 for combination therapy vs. monotherapy groups. Error bars: ± 2 SEM.

To further assess the therapeutic efficacy of combined AR and MEK inhibition in molecular apocrine breast cancer, we generated xenograft tumors using MDA-MB-453 cell line. This cell line was chosen for the xenograft studies because it is a prototype of molecular apocrine subtype and has been previously employed for *in vivo *studies of the AR-ERK feedback loop [4,5,9,12]. PD0325901 treatment was carried out at 5 mg/kg/day based on the results of our toxicity studies. Mouse treatments were carried out in the following four groups: (1) placebo pellet and daily oral gavage of carrier solution (control group), (2) flutamide 25 mg/60 days pellet + gavage of carrier solution (flutamide monotherapy), (3) daily oral gavage of PD0325901 at 5 mg/kg/day + placebo pellet (PD0325901 monotherapy) and (4) flutamide pellet + PD0325901 (combination therapy). Six mice were treated in each experimental group for 30 days, and fold change in tumor volume was calculated as described in Materials and methods. We observed a threefold lower tumor volume change in the combination therapy group compared to that of control (*P *< 0.01, Figure 6A). Importantly, mice treated with combination therapy had approximately 2.5-fold lower tumor growth compared to that of monotherapy groups (*P *< 0.01, Figure 6A and 6B).

**Figure 7:**
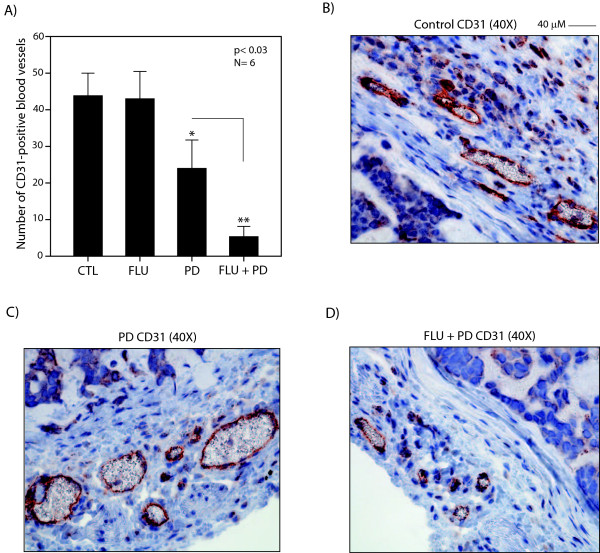
**The therapeutic effect of AR and MEK inhibitors on *in vivo *angiogenesis**. **(A) **Angiogenesis index for each *in vivo *treatment group. Angiogenesis was measured as the number of CD-31-positive blood vessels in a cross-section of each xenograft tumor. CTL: control group; FLU: flutamide; and PD: PD0325901. *P < 0.03 for PD0325901 monotherapy vs. control and **P < 0.03 for combination therapy vs. monotherapy groups using Mann-Whitney *U *test. Error bars: ± 2 SEM. **(B) **Immunohistochemistry (IHC) was used to measure angiogenesis in a control xenograft tumor. Staining was performed using a CD31 rabbit polyclonal antibody. Original magnification, × 40. **(C) **IHC was used to measure angiogenesis in a PD0325901 monotherapy tumor. Original magnification, × 40. **(D) **IHC was used to measure angiogenesis in a xenograft tumor treated with combination therapy. Original magnification, × 40.

We next investigated the effect of different *in vivo *treatments on cellular proliferation and angiogenesis using harvested xenograft tumors. Proliferation index and angiogenesis were assessed with IHC using Ki-67 and CD31 antibodies, respectively. The results were then compared between different *in vivo *therapy groups. Notably, we observed a proliferation index of 22% ± 2 in tumors treated with the combination therapy, which was significantly lower than that of control (56% ± 2) and monotherapy groups (flutamide: 39% ± 3, PD0325901: 31% ± 4), (*P *< 0.05, Figure 6C to 6E). Furthermore, angiogenesis was significantly lower in the combination therapy group with a CD31-positive blood vessel count of 5.3 ± 3 compared to that of control (44 ± 6) and monotherapy groups (flutamide: 43 ± 7, PD0325901: 24 ± 7) (*P *< 0.03, Figure 7A to 7D). Moreover, CD-31-positive blood vessels in the combination therapy group were smaller and less distinct than those in other groups (Figure 7B to 7D).

These findings indicate that the combination therapy with fluatmide and PD0325901 has a significantly higher level of *in vivo *activity in the reduction of xenograft tumor growth, cellular proliferation and angiogenesis compared to that of monotherapies with these agents. It is also notable that flutamide and PD0325901 monotherapies did not significantly reduce tumor growth compared to the control group (Figure 6A and 7A). Therefore, a significantly higher efficacy in the combination therapy group compared to that of monotherapies suggests an *in vivo *synergy between fluatmide and PD0325901.

### Synergy between AR and MEK inhibitors overcomes trastuzumab resistance

**Figure 8:**
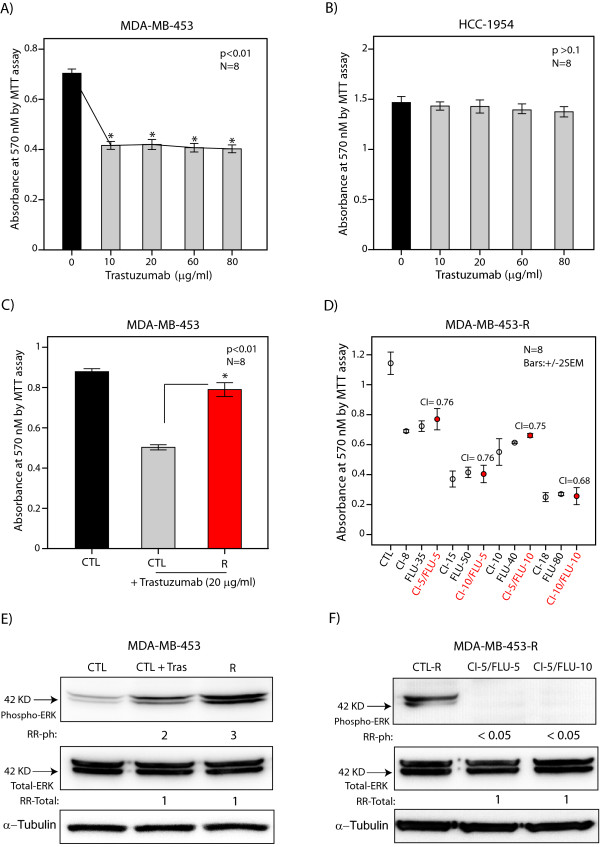
**Synergy between AR and MEK inhibitors in trastuzumab-resistant cells**. **(A) **MTT assay was used to measure cell viability in MDA-MB-453 cell line following trastuzumab treatment at 10 to 80 μg/ml concentrations. CTL: control. *P < 0.01 for trastuzumab groups vs. control. Error bars: ± 2 SEM. **(B) **Cell viability in HCC-1954 cell line following trastuzumab treatment as described in Figure 8A. **(C) **Cell viability in trastuzumab-resistant MDA-MB-453 (R) compared to the untreated control and control line treated with trastuzumab at 20 μg/ml. *P < 0.01 for R vs. treated control cells. **(D) **Combination indices (CI) for flutamide (FLU) and CI-1040 combination therapy in MDA-MB-453-R line using MTT assay. Therapies were carried out with flutamide at 5 and 10 μM with each concentration of CI-1040 at 5 and 10 μM (CI-5 and CL-10). The concentrations of fluatmide and CI-1040 monotherapies with an effect similar to that of each combination therapy are depicted. Error bars: ± 2 SEM. **(E) **Western blot analysis was used to measure the phosphorylated (ph) and total ERK levels in MDA-MB-453-R, control MDA-MB-453 and control MDA-MB-453 treated with trastuzumab at 20 μg/ml concentration (CTL + Tras). Fold changes in band densities (RR) were measured relative to the control. **(F) **Western blot analysis was used to measure the phosphorylated and total ERK levels in MDA-MB-453-R line following combination therapies with CI-1040 (5 μM)/flutamide (5 μM) and CI-1040 (5 μM)/flutamide (10 μM). RR values were measured relative to the untreated MDA-MB-453-R line.

It is known that at least 50% of ER-/AR+ breast tumors have ErbB2 overexpression, and anti-ErbB2 treatment is an established part of management for this subgroup [7,8,25]. Importantly, trastuzumab resistance is a major clinical problem in this patient population [26]. Therefore, we investigated the activity of combination therapy with flutamide and CI-1040 in overcoming trastuzumab resistance using molecular apocrine cell lines MDA-MB-453 and HCC-1954 with known ErbB2 overexpression [5,9]. We first examined the effect of trastuzumab treatment at 10 to 80 μg/ml concentrations for 48 hours on cell viability of MDA-MB-453 and HCC-1954 lines using MTT assay. A solvent-only-treated group was used as control. We observed a significant reduction in cell viability by approximately 40% following trastuzumab treatments in MDA-MB-453 cell line (*P *< 0.01, Figure 8A). In addition, trastuzumab activity reached a plateau at 10 μg/ml concentration without any additional reduction in cell viability at higher concentrations of this agent (Figure 8A). Furthermore, HCC-1954 cell line showed an intrinsic resistance to trastuzumab treatment with no significant reduction in cell viability at any of the tested concentrations (Figure 8B).

Next, we generated a trastuzumab-resistant MDA-MB-453 line (MDA-MB-453-R) as described in Materials and methods. We confirmed that MDA-MB-453-R cells are resistant to trastuzumab at 20 μg/ml concentration using MTT assay. MDA-MB-453-R line showed a level of cell viability in the presence of trastuzumab similar to that observed in untreated control line (Figure 8C). In contrast, the control line demonstrated a significant reduction in cell viability following trastuzumab treatment at 20 μg/ml concentration for 48 hours (*P *< 0.01, Figure 8C). Subsequently, we calculated CI values to assess synergy between flutamide and CI-1040 in MDA-MB-453-R line. Flutamide and CI-1040 treatments were carried out at the same four dose combinations applied before in the nonresistant line (CI-1040 (5 μM)/flutamide (5 μM), CI-1040 (10 μM)/flutamide (5 μM), CI-1040 (5 μM)/flutamide (10 μM), and CI-1040 (10 μM)/flutamide (10 μM)). Importantly, we observed a synergy at all four dose combinations in MDA-MB-453-R line with CI values of 0.68 to 0.76 (Figure 8D).

The synergy between flutamide and CI-1040 in MDA-MB-453-R line raises the possibility of a functional role for ERK phosphorylation in the process of trastuzumab resistance in molecular apocrine cells. To investigate this possibility, we assessed the level of phosphorylated and total ERK proteins in untreated MDA-MB-453 control, MDA-MB-453 control treated with trastuzumab at 20 μg/ml, and MDA-MB-453-R cell lines. Importantly, MDA-MB-453-R line showed a threefold higher level of ERK phosphorylation compared to that of untreated control (Figure 8E). In addition, there was an induction of ERK phosphorylation by twofold following trastuzumab treatment for 48 hours in the control line (Figure 8E). It is notable that there was no difference between the levels of total ERK across these experiments (Figure 8E). Moreover, combination therapies with CI-1040 (5 μM)/flutamide (5 μM) and CI-1040 (5 μM)/flutamide (10 μM) completely abrogated ERK phosphorylation in MDA-MB-453-R line (Figure 8F). Taken together, these data suggest that the synergy between flutamide and CI-1040 can overcome trastuzumab resistance in molecular apocrine cells. In addition, this combination therapy abrogates the induction of ERK phosphorylation observed in trastuzumab-resistant cells.

## Discussion

Management of ER-breast cancer is challenging due to the limited therapeutic targets available in this disease. Heterogeneity of ER-breast cancer contributes to this challenge, and therefore identification of novel targeted therapies requires a robust biological understanding of different ER-subtypes. We have recently identified a positive feedback loop between the AR and ERK signaling pathways in molecular apocrine subtype of ER-breast cancer [12]. In this process, AR regulates ERK phosphorylation and kinase activity as well as the phosphorylation of ERK target proteins RSK1 and Elk-1 [12]. Notably, AR inhibition using flutamide abrogates ERK phosphorylation in a dose-dependent manner, and AR activation using DHT leads to an increase in ERK phosphorylation mediated through ErbB2 [12]. In turn, ERK signaling regulates AR expression mediated through transcription factor CREB1 [12].

In this study, we explored the therapeutic implications of the AR-ERK feedback loop in molecular apocrine breast cancer. This was investigated using the combination therapy with AR and MEK inhibitors, which are clinically available and constitute effective targeted therapies to block the AR and ERK signaling pathways, respectively [14,16]. We applied CI-1040 and PD0325901 for *in vitro *and *in vivo *inhibition of MEK, respectively. This approach was used due to the fact that CI-1040 has been commonly used to study the effect of MEK inhibitors on cell lines and PD0325901 is a derivative of CI-1040 with a better oral bioavailability, which makes this agent more suitable for *in vivo *studies [19,21-23].

Importantly, we demonstrated synergistic CI values for the combination therapy with AR inhibitor flutamide and MEK inhibitor CI-1040 across three molecular apocrine cell lines (Figures 1 to 4 and Table 1). Furthermore, this synergy was present at four dose combinations in each cell line using both cell viability and apoptosis assays, suggesting a reproducible synergy between flutamide and CI-1040 in molecular apocrine cells. Moreover, we showed *in vivo *that the combination therapy with flutamide and MEK inhibitor PD0325901 has a significantly higher therapeutic efficacy in reducing tumor growth, cellular proliferation and angiogenesis compared to monotherapies with these agents in a xenograft molecular apocrine model (Figures 6 and 7).

A combination therapy approach provides an attractive option in the management of ER-/AR+ breast cancer, since it exploits the synergy between AR and MEK inhibitors and at the same time minimizes their potential toxicities by requiring a lower dose of each agent in the combination setting. This is particularly relevant for MEK inhibitors, as higher doses of these drugs have been associated with significant toxicities in clinical trials [27-29]. In fact, our *in vivo *data clearly demonstrated that higher doses of PD0325901 have toxicity in mice, and this was absent at the 5 mg/kg/day dose used for the combination therapy studies (Figure 5). Another advantage of using lower doses of PD0325901 and flutamide in xenograft studies is to show an *in vivo *synergy between AR and MEK inhibitors. A similar approach has been previously applied to assess *in vivo *synergy for other agents [30,31]. Notably, we observed that monotherapies did not significantly reduce tumor growth in mice, and therefore a markedly lower tumor growth with the combination therapy compared to that of control and monotherapy groups suggests an *in vivo *synergy between flutamide and PD0325901 (Figures 6A and 7A).

The AR-ERK positive feedback loop forms the molecular basis for the synergy observed between AR and MEK inhibitors [12]. This is supported by the fact that flutamide synergistically enhances the effect of MEK inhibitor CI-1040 in reducing the level of ERK phosphorylation in molecular apocrine cells [12]. In addition, CI-1040 treatment results in a reduction of AR expression in molecular apocrine cell lines [12]. Furthermore, we have previously shown a synergy between flutamide and Cdc25A inhibitor PM-20 in molecular apocrine cells that was associated with a decrease in the phosphorylation levels of ERK target proteins RSK1 and Elk-1 [9]. Therefore, cross-regulation between the AR and ERK signaling pathways provides an attractive therapeutic target in molecular apocrine breast cancer. Moreover, a number of potent second-generation AR inhibitors such as abiraterone and MDV3100 are currently being studied in androgen-refractory prostate cancer [32,33]. Since there is growing evidence to support the role of AR as a target for therapy in molecular apocrine breast cancer, the new AR inhibitors may potentially provide additional treatment options in the management of this disease.

ErbB2 amplification and overexpression are present in at least 50% of molecular apocrine tumors, and the affected patients are usually started on trastuzumab early in the course of their disease [7,8,25]. However, there is a high rate of intrinsic resistance to trastuzumab monotherapy among patients with ErbB2-positive breast cancer, ranging from 66% to 88% [26,34]. Furthermore, patients with a primary response to trastuzumab monotherapy have a short median time to progression of only 4.9 months [35]. As a result, trastuzumab monotherapy is commonly combined with chemotherapy agents to increase response rates and time to disease progression; however, this approach is associated with more side effects [35,36]. In this study, we demonstrated that flutamide and CI-1040 combination leads to a synergistic reduction of cell viability in HCC-1954 and MDA-MB-453-R cell lines with intrinsic and acquired resistance to trastuzumab, respectively (Figures 2C and 8A to 8D). Therefore, combination therapy with AR and MEK inhibitors may provide an effective treatment option in ErbB2-positive molecular apocrine patients with trastuzumab resistance.

A number of different mechanisms have been proposed for trastuzumab resistance, including compensatory signaling and altered downstream signaling [26,37,38]. We found an increased level of ERK phosphorylation shortly after trastuzumab treatment in molecular apocrine cells (Figure 8E). This effect on ERK phosphorylation following acute exposure to trastuzumab has been reported in other ErbB2-positive cell lines and is similar to MAPK/ERK activation in cells stimulated with exogenous ErbB ligands [39,40]. Importantly, we observed that the level of ERK phosphorylation further increased in trastuzumab-resistant MDA-MB-453-R cell line, which was abrogated following flutamide and CI-1040 combination therapy (Figures 8E and 8F). These findings are in agreement with the previous reports that trastuzumab-resistant cells are exquisitely sensitive to MEK inhibition [41]. Therefore, the observed induction of ERK in trastuzumab-resistant molecular apocrine cells may render these cells dependent on MAPK/ERK signaling and sensitizes them to the synergy between AR and MEK inhibitors.

## Conclusions

In this study, we investigated the AR-ERK feedback loop as a therapeutic target in molecular apocrine breast cancer and demonstrated *in vitro *and *in vivo *synergies between AR and MEK inhibitors in this subtype. Furthermore, we showed that the combination therapy with these inhibitors can overcome trastuzumab resistance in molecular apocrine cells. Therefore, a combination therapy strategy with AR and MEK inhibitors may provide an attractive therapeutic option for molecular apocrine breast cancer. Future clinical trials are required to test the application of this approach in patient management.

## Abbreviations

DHT: dihydrotestosterone; ERK: extracellular signal-regulated kinase; MAPK: mitogen-activated protein kinase; MEK: mitogen-activated protein kinase kinase.

## Competing interests

AN is named on a patent application related to the content of this manuscript. All other authors declare that they have no competing interests.

## Authors' contributions

AN conceived the study, designed the experiments and drafted the manuscript. AN, KMC and JL carried out the experiments. All authors read and approved the final manuscript.

## References

[B1] Putti TC El-Rehim DM Rakha EA Paish CE Lee AH Pinder SE Ellis IO Estrogen receptor-negative breast carcinomas: a review of morphology and immunophenotypical analysis Mod Pathol 2005 18 26 35 10.1038/modpathol.3800255 15332092

[B2] Farmer P Bonnefoi H Becette V Tubiana-Hulin M Fumoleau P Larsimont D Macgrogan G Bergh J Cameron D Goldstein D Duss S Nicoulaz AL Brisken C Ficke M Delorenzi M Iggo R Identification of molecular apocrine breast tumours by microarray analysis Oncogene 2005 24 4660 4671 10.1038/sj.onc.1208561 15897907

[B3] Teschendorff AE Naderi A Barbosa-Morais NL Caldas C PACK: Profile Analysis using Clustering and Kurtosis to find molecular classifiers in cancer Bioinformatics 2006 22 2269 2275 10.1093/bioinformatics/btl174 16682424

[B4] Doane AS Danso M Lal P Donaton M Zhang L Hudis C Gerald WL An estrogen receptor-negative breast cancer subset characterized by a hormonally regulated transcriptional program and response to androgen Oncogene 2006 25 3994 4008 10.1038/sj.onc.1209415 16491124

[B5] Naderi A Hughes-Davies L A functionally significant cross-talk between androgen receptor and ErbB2 pathways in estrogen receptor negative breast cancer Neoplasia 2008 10 542 548 18516291 10.1593/neo.08274 PMC2386539

[B6] Niemeier LA Dabbas DJ Beriwal S Striebel JM Bhargava R Androgen receptor in breast cancer: expression in estrogen receptor-positive tumors and in estrogen receptor-negative tumors with apocrine differentiation Mod Pathol 2009 23 205 212 10.1038/modpathol.2009.159 19898421

[B7] Park S Koo J Park HS Kim JH Choi SY Lee JH Park BW Lee KS Expression of androgen receptors in primary breast cancer Ann Oncol 2010 21 488 492 19887463 10.1093/annonc/mdp510

[B8] Vranic S Tawfik O Palazzo J Bilalovic N Eyzaguirre E Lee LMJ Adegboyega P Hagenkord J Gatalica Z EGFR and HER-2/neu expression in invasive apocrine carcinoma of the breast Mod Pathol 2010 23 644 653 10.1038/modpathol.2010.50 20208479

[B9] Naderi A Liu J Inhibition of androgen receptor and Cdc25A phosphatase as a combination targeted therapy in molecular apocrine breast cancer Cancer Lett 2010 298 74 87 10.1016/j.canlet.2010.06.005 20605569

[B10] Traina TA Feigin K Patil S Yuan J Dickler M D'Andrea G Bromberg J Hudis C Androgen receptor inhibition can stabilize disease in patients with AR^-^, ER^-^/PR^- ^metastatic breast cancer Ann Oncol 2009 20 ii63 ii64

[B11] Sanga S Broom BM Cristini V Edgerton ME Gene expression meta-analysis supports existence of molecular apocrine breast cancer with a role for androgen receptor and implies interactions with ErbB family BMC Med Genomics 2009 2 59 10.1186/1755-8794-2-59 19747394 PMC2753593

[B12] Chia KM Liu J Francis GD Naderi A A feedback loop between androgen receptor and ERK signaling in estrogen receptor-negative breast cancer Neoplasia 2011 13 154 166 21403841 10.1593/neo.101324 PMC3033594

[B13] Levine D Park K Juretzka M Esch J Hensley M Aghajanian C Lewin S Konner J Derosa F Spriggs D Lasonos A Sabbatini P A phase II evaluation of goserelin and bicalutamide in patients with ovarian cancer in second or higher complete clinical disease remission Cancer 2007 110 2448 2456 10.1002/cncr.23072 17918264

[B14] Singh SM Gauthier S Labrie F Androgen receptor antagonists (antiandrogens): structure-activity relationships Curr Med Chem 2000 7 211 247 10637363 10.2174/0929867003375371

[B15] Friday BB Adjei AA Advances in targeting the Ras/Raf/MEK/Erk mitogen-activated protein cascade with MEK inhibitors for cancer therapy Clin Cancer Res 2008 14 342 346 10.1158/1078-0432.CCR-07-4790 18223206

[B16] McCubery JA Steelman LS Abrams SL Chappell W Russo S Ove R Miella M Tafuri A Lunghi P Bonatli A Stivala F Nicoletti F Libra M Martelli AM Montalto G Cervello M Emerging MEK inhibitors Expert Opin Emerg Drugs 2010 15 203 223 10.1517/14728210903282760 20151845

[B17] Naderi A Hughes-Davies L Nerve growth factor/nuclear factor-κB pathway as a therapeutic target in breast cancer J Cancer Res Clin Oncol 2009 135 211 216 10.1007/s00432-008-0455-6 18716795 PMC12160292

[B18] Zhao L Wientjes MG Au JLS Evaluation of combination chemotherapy: integration of nonlinear regression, curve shift, isobologram, and combination index analyses Clin Cancer Res 2004 10 7994 8004 10.1158/1078-0432.CCR-04-1087 15585635

[B19] Hoeflich KP O'Brien C Boyd Z Cavet G Guerrero S Jung K Januario T Savage H Punnoose E Truong T Zhou W Berry L Murray L Amler L Belvin M Friedman LS Lackner MR In vivo antitumor activity of MEK and phosphatidylinositol 3-kinase inhibitors in basal-like breast cancer models Clin Cancer Res 2009 15 4649 4664 10.1158/1078-0432.CCR-09-0317 19567590

[B20] Lehnes K Winder AD Alfonso C Kasid N Simoneaux M Summe H Morgan E Iann MC Duncan J Eagan M Tavaluc R Evans CH Jr Russel R Wang A Hu F Stoica A The effect of estradiol on in vivo tumorigenesis is modulated by the human epidermal growth factor receptor 2/phosphatidylinositol 3-kinase/Akt1 pathway Endrocrinology 2007 148 1171 1180 10.1210/en.2006-1179 17138652

[B21] Liu D Liu Z Jiang D Dackiw AP Xing M Inhibitory effects of the mitogen-activated protein kinase kinase inhibitor CI-1040 on the proliferation and tumor growth of thyroid cancer cells with BRAF or RAS mutations J Clin Endocrinol Metab 2007 92 4686 4695 10.1210/jc.2007-0097 17911174

[B22] Sambade MJ Camp JT Kimple RJ Sartor CI Shields JM Mechanism of lapatinib-mediated radiosensitization of breast cancer cells is primarily by inhibition of the Raf>MEK>ERK mitogen-activated protein kinase cascade and radiosensitization of lapatinib-resistant cells restored by direct inhibition of MEK Radiother Oncol 2009 93 639 644 10.1016/j.radonc.2009.09.006 19853943 PMC2799330

[B23] Solit DB Garraway LA Pratilas CA Sawai A Getz G Basso A Ye Q Lobo JM She Y Osman I Golub TR Sebolt-Leopold J Sellers WR Rosen N BRAF mutation predicts sensitivity to MEK inhibition Nature 2006 439 358 362 10.1038/nature04304 16273091 PMC3306236

[B24] Barrett SD Bridges AJ Dudely DT Saltiel AR Fergus JH Flamme CM Delaney AM Faufman M LePage S Leopold WR Przybranowski SA Sebolt-Leopold J Van Becelaere K Doherty AM Kennedy RM Marston D Howard WAJ Smith Y Warmus JS Tecle H The discovery of the benzhydroxamate MEK inhibitors CI-1040 and PD 0325901 Bioorg Med Chem Lett 2008 18 6501 6504 10.1016/j.bmcl.2008.10.054 18952427

[B25] Zhao JJ Silver DP Estrogen receptor-negative breast cancer: new insights into subclassification and targeting Clin Cancer Res 2009 15 6327 6340 10.1158/1078-0432.CCR-09-1107 19825953 PMC2904516

[B26] Nahta R Esteva FJ HER2 therapy: molecular mechanisms of trastuzumab resistance Breast Cancer Res 2006 8 215 10.1186/bcr1612 17096862 PMC1797036

[B27] Sebolt-Leopold JS Advances in the development of cancer therapeutics directed against the RAS-mitogen-activated protein kinase pathway Clin Cancer Res 2008 14 3651 3656 10.1158/1078-0432.CCR-08-0333 18559577

[B28] LoRusso PM Kirshnamurthi SS Rinehart JJ Nabell LM Malburg L Chapman PB DePrimo SE Bentivegna S Wilner KD Tan W Ricart AD Phase I pharmacokinetic and pharmacodynamic study of the oral MAPK/ERK kinase inhibitor PD-0325901 in patients with advanced cancers Clin Cancer Res 2010 16 1924 1937 10.1158/1078-0432.CCR-09-1883 20215549

[B29] Adjei AA Cohen RB Franklin W Morris C Wilson D Molina JR Hanson LJ Gore L Chow L Leong S Maloney L Gordon G Simmons H Marlow A Litwiler K Brown S Poch G Kane K Haney J Eckhardt SG Phase I pharmacokinetic and pharmacodynamic study of the oral, small-molecule mitogen-activated protein kinase kinase 1/2 inhibitor AZD6244 (ARRY-142886) in patients with advanced cancers J Clin Oncol 2008 26 2139 2146 10.1200/JCO.2007.14.4956 18390968 PMC2718422

[B30] Marchion DC Bicaku E Daud AI Sullivan DM Munster PN In vivo synergy between topoisomerase II and histone deacetylase inhibitors: predictive correlates Mol Cancer Ther 2005 4 1993 2000 10.1158/1535-7163.MCT-05-0194 16373714

[B31] Skobeleva N Menon S Weber L Golemis EA Khazak V In vitro and in vivo synergy of MCP compounds with mitogen-activated protein kinase pathway- and microtubule-targeting inhibitors Mol Cancer Ther 2007 6 898 906 10.1158/1535-7163.MCT-06-0602 17363484 PMC2670615

[B32] Hsieh AC Ryan CJ Novel concepts in androgen receptor blockade Cancer J 2008 14 11 14 10.1097/PPO.0b013e318161d13e 18303477

[B33] Tran C Ouk S Clegg NJ Chen Y Watson PA Arora V Wongvipat J Smith-Jones PM Yoo D Kwon A Wasielewska T Welsbie D Chen CD Higano CS Beer TM Hung DT Scher HI Jung ME Sawyers CL Development of a second-generation antiandrogen for treatment of advanced prostate cancer Science 2009 324 787 790 10.1126/science.1168175 19359544 PMC2981508

[B34] Cobleigh MA Vogel CL Tripathy D Robert NJ Scholl S Fehrenbacher L Wolter JM Paton V Shak S Lieberman G Slamon DJ Multinational study of the efficacy and safety of humanized anti-HER2 monoclonal antibody in women who have HER2-overexpressing metastatic breast cancer that has progressed after chemotherapy for metastatic disease J Clin Oncol 1999 17 2639 2648 10561337 10.1200/JCO.1999.17.9.2639

[B35] Slamon DJ Leyland-Jones B Shak S Fuchs H Paton V Bajamonde A Fleming T Eiermann W Wolter J Pegram M Baselga J Norton L Use of chemotherapy plus a monoclonal antibody against HER2 for metastatic breast cancer that overexpresses HER2 N Engl J Med 2001 344 783 792 10.1056/NEJM200103153441101 11248153

[B36] Seidman AD Fornier MN Esteva FJ Tan L Kaptain S Bach A Panageas KS Arroyo C Valero V Currie V Gilewski T Theodoulou M Moynahan ME Moasser M Sklarin N Dickler M D'Andrea G Cristofanilli M Rivera E Hortobagyi GN Norton L Hudis CA Weekly trastuzumab and paclitaxel therapy for metastatic breast cancer with analysis of efficacy by HER2 immunophenotype and gene amplification J Clin Oncol 2001 19 2587 2595 11352950 10.1200/JCO.2001.19.10.2587

[B37] Lu Y Zi X Pollak M Molecular mechanisms underlying IGF-I-induced attenuation of the growth-inhibitory activity of trastuzumab (Herceptin) on SKBR3 breast cancer cells Int J Cancer 2004 108 334 341 10.1002/ijc.11445 14648698

[B38] Yakes FM Chinratanalab W Ritter CA King CA Seelig S Arteaga CL Herceptin-induced inhibition of phosphatidylinositol-3 kinase and Akt Is required for antibody-mediated effects on p27, cyclin D1, and antitumor action Cancer Res 2002 62 4132 4141 12124352

[B39] Gijsen M King P Perera T Parker PJ Harris AL Larijani B Kong A HER2 phosphorylation is maintained by a PKB negative feedback loop in response to anti-HER2 herceptin in breast cancer PLoS Biol 2010 8 e1000563 10.1371/journal.pbio.1000563 21203579 PMC3006345

[B40] Kiyatkin A Aksamitiene E Markevich NI Borisov NM Hoek JB Kholodenko BN Scaffolding protein Grb2-associated binder 1 sustains epidermal growth factor-induced mitogenic and survival signaling by multiple positive feedback loops J Biol Chem 2006 281 19925 19938 10.1074/jbc.M600482200 16687399 PMC2312093

[B41] Zhuang G Brantley-Sieders DM Vaught D Yu J Xie L Wells S Jackson D Muraoka-Cook R Arteaga C Chen J Elevation of receptor tyrosine kinase EphA2 mediates resistance to trastuzumab therapy Cancer Res 2010 70 299 308 10.1158/0008-5472.CAN-09-1845 20028874 PMC3859619

